# Management of hypocalcaemia in the critically ill

**DOI:** 10.1097/MCC.0000000000001059

**Published:** 2023-07-03

**Authors:** Max Melchers, Arthur Raymond Hubert van Zanten

**Affiliations:** a Department of Intensive Care Medicine, Gelderse Vallei Hospital, Ede; b Division of Human Nutrition and Health, Wageningen University & Research, HELIX (Building 124), Wageningen, the Netherlands

**Keywords:** calcium, hypotension, mortality, sepsis, shock

## Abstract

To better understand the established associations between hypocalcaemia and clinical outcomes, we synopsize the mechanisms involved in hypocalcaemia in the critically ill. We also provide an overview of the current evidence on managing hypocalcaemia in critical illness.

**Recent findings:**

Hypocalcaemia is reported to occur in 55–85% of ICU patients. It appears to be associated with poor outcomes, but it may be a marker rather than a direct cause of disease severity. The recommendations to correct calcium in major bleeding are found on weak evidence and require further exploration by a randomized controlled trial (RCT). Calcium administration in cardiac arrest has shown no benefit and may provoke harm. In addition, no RCT has assessed the risks and benefits of calcium supplementation in critically ill hypocalcemic patients. Several recent studies conclude that it may even harm septic ICU patients. These observations are supported by evidence that septic patients using calcium channel blockers may have better outcomes.

**Summary:**

Hypocalcaemia is common in critically ill patients. Direct evidence that calcium supplementation improves their outcomes is lacking, and there is even some indication that it may be detrimental. Prospective studies are required to elucidate the risks and benefits, and the pathophysiological mechanisms involved.

## INTRODUCTION

There is no doubt that critically ill patients very frequently have low extracellular calcium levels and possibly high intracellular concentrations [1,2]. Serum hypocalcaemia appears to be associated with poorer outcome, which may lead to physicians attempting to correct hypocalcaemia by administering parenteral calcium to critically ill patients without an evidence-based threshold [1,3]. Several recently published studies provide fascinating new insights into managing hypocalcaemia in the ICU, questioning whether it is desirable to supplement calcium in the critically ill. This review provides an updated summary of the pathophysiologic mechanisms involved in hypocalcaemia alongside its consequences for the critically ill and focuses on the risks and benefits of managing hypocalcaemia in the ICU. 

**Box 1:**
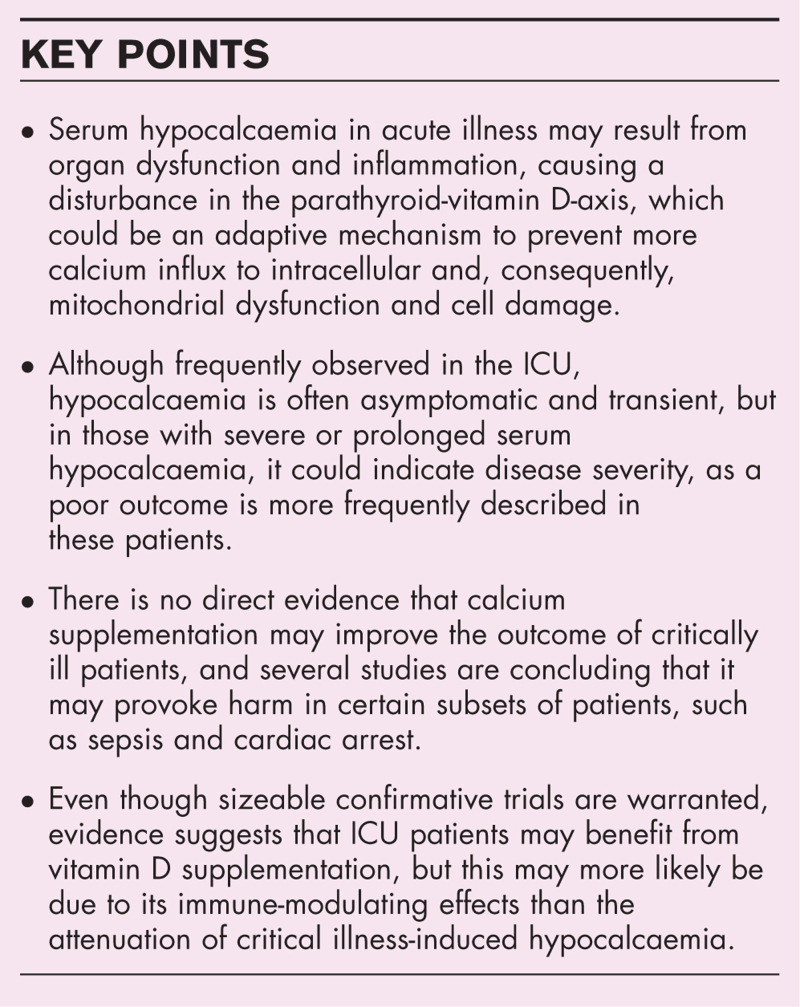
no caption available

## CALCIUM HOMEOSTASIS IN HEALTH AND CRITICAL ILLNESS

Calcium is an essential ion with numerous vital physiological roles in the human body, including cell-to-cell signalling, membrane potential, neurotransmission and blood coagulation [1,2,4]. Nearly all is stored in the bone, and a mere 1% resides in the extracellular component [2].

### Intracellular

Calcium is a versatile intracellular second messenger involved in complex cellular mechanisms, including differentiation, contraction, mitosis and cell death [5]. In resting and healthy conditions, the calcium levels in the cytoplasm and mitochondrial matrix are tightly kept about 20 000 times lower than in the extracellular milieu under the influence of plasma membrane transporters and storage in organelles other than the mitochondria [5–8]. Extrinsic stressors or cell stimuli, such as membrane depolarisation or extracellular signalling molecules, may temporarily increase cytoplasmic calcium by influx from extracellular or release from intracellular stores to interact with intracellular proteins [5,8]. During a transient increase in cytoplasmatic calcium, mitochondria utilize calcium as a signal for energy demand by activating adenosine triphosphate synthase and stimulating the electron transport chain to regulate reactive oxygen species production [8–11]. However, severe extrinsic stressors, such as cell damage, oxidative stress or inflammation, will cause excessive cytosolic calcium, which will be buffered in the cell's mitochondria [8,10]. Excessive calcium in the mitochondria damages the organelle and causes leakage of mitochondrial components into the circulation leading to uncontrolled autophagy and additional cell damage [8,12^▪^]. This effect will induce even more calcium influx into the cell, eventually leading to apoptosis by calcium-activated calpains and caspase proteins [8,10]. Aside from cell damage mediated calcium influx, mitochondrial dysfunctioning primarily could also be underlying high intracellular calcium, as cytosolic calcium concentrations are maintained low by calcium-ATP-ase pumps [8,11]. Energy failure due to critical illness induced mitochondrial dysfunctioning may hereby contribute to cytosolic calcium overload [8,12^▪^]. Numerous studies have shown that intracellular calcium is elevated in various severe illnesses in animal models, especially when suffering from excessive inflammation [13–17].

### Extracellular

**FIGURE 1:**
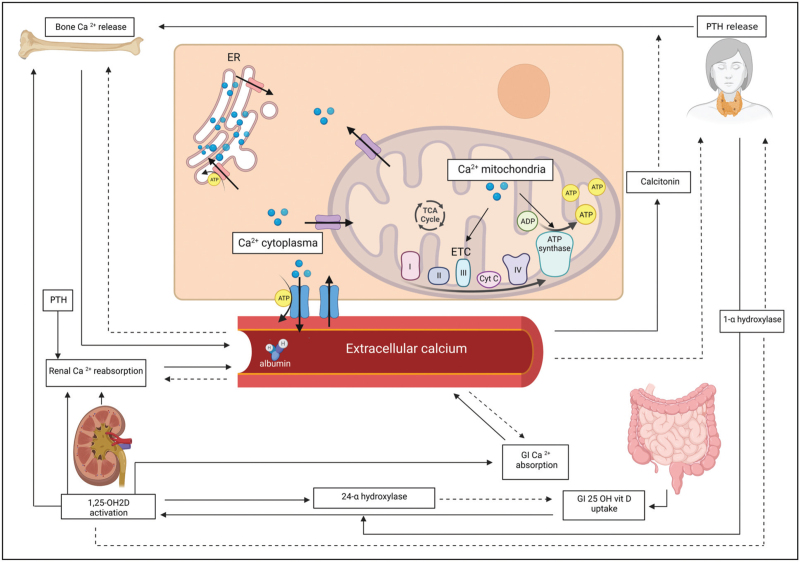
Schematic diagram depicting intracellular and extracellular calcium homeostasis in health. Arrow = stimulating action; dashed arrow = inhibiting action. 25-OH vit D, 25-hydroxyvitamin D; Ca ^2+^, calcium; ER, endoplasmatic reticulum; ETC, electron transport chain; GI, gastrointestinal; PTH, parathyroid hormone; TCA cycle, tricarboxylic acid/citric acid cycle. Figure created with BioRender.com.

The extracellular calcium levels are maintained within normal ranges primarily under the influence of the parathyroid hormone (PTH) and vitamin D, and to a lesser extent, calcitonin, making calcium homeostasis closely related to that of phosphate and magnesium (Fig. 1) [2,4,18,19]. A decrease in serum calcium can be induced by hypomagnesemia and/or hyperphosphatemia by reducing PTH secretion and activity [18]. The extracellular calcium fraction is measured in serum, wherein in a healthy state, less than half of the calcium is buffered by negatively charged amino acids, such as albumin and globulins, and a small portion is a component in complexes [4]. Roughly half of the extracellular calcium exists as ionized calcium (iCa), the biologically active form responsible for most of the aforementioned physiological functions [1,2,4]. iCa is susceptible to acid-base status changes as hydrogen ions compete with calcium for binding sites on albumin [2,4]. An acidic pH will increase iCa, while alkalosis has the opposite effect. Hence, serum calcium is subject to alterations in circulating proteins, acid-base status and electrolyte levels commonly observed in critically ill patients. Therefore, measuring iCa in the ICU is generally advised instead of using formulas to correct the total calcium level [1].

**FIGURE 2:**
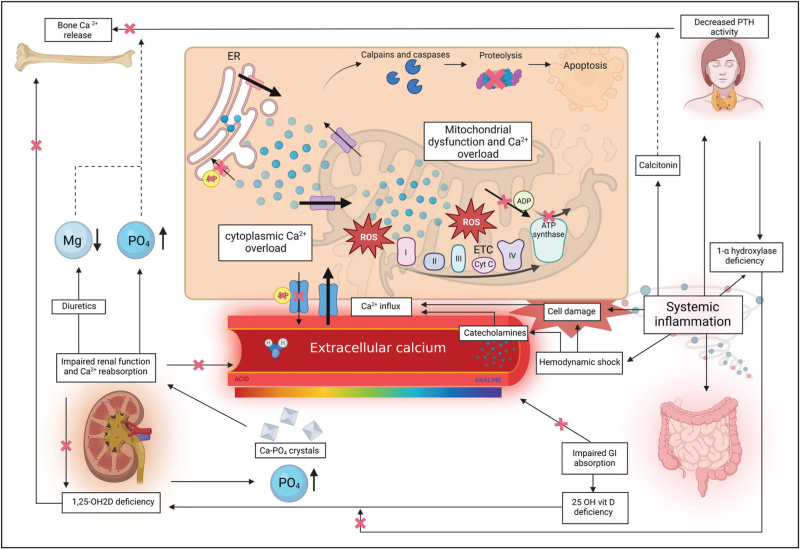
Schematic diagram depicting the proposed pathophysiological mechanisms involved in extracellular hypocalcaemia and high intracellular calcium during critical illness. Arrow = stimulating action; dashed arrow = inhibiting action; arrow with cross = inhibited action. 25-OH vit D, 25-hydroxyvitamin D; Ca ^2+^, calcium; ER, endoplasmatic reticulum; ETC, electron transport chain; GI, gastrointestinal; Mg, magnesium; PO_4_, phosphate; PTH, parathyroid hormone; ROS, reactive oxygen species. Figure created with BioRender.com.

Extracellular calcium homeostasis is also altered in critically ill patients due to systemic inflammation and impaired organ functioning [1,2]. Circulating cytokines cause an increase in calcitonin secretion and a decrease in PTH sensitivity [1,2,20,21]. Furthermore, impaired renal functioning may lead to decreased calcium reabsorption in the tubules, reduced activation of vitamin D(1,25(OH)2D) and impaired phosphate clearance, which directly and indirectly lowers extracellular calcium levels [1,2]. Gastrointestinal dysfunction is frequently encountered in critically ill patients, which may cause impaired nutrient absorption, including calcium and 25-hydroxyvitamin D [22^▪^]. Iatrogenic causes of hypocalcemia during ICU admission include medications such as diuretics and catecholamines and infusion with phosphate and citrate such as citrate-containing blood products or as anticoagulation during continuous renal replacement therapy (CRRT) [1,2,23]. All these mechanisms make the critically ill patient prone to ionized hypocalcaemia (Fig. 2).

## THE CONSEQUENCES OF HYPOCALCAEMIA IN THE CRITICALLY ILL

Although ionized hypocalcaemia appears to be very prevalent among patients admitted to the ICU, the classical symptoms related to lowered calcium levels, such as tetany, seizures, prolonged QT and cardiac dysfunction, are uncommonly described in the ICU patient because they are generally asymptomatic, mild or may be concealed by the aspects of acute illness [1,2,23,24]. The laboratory definition of hypocalcaemia varies by institution. However, it is generally defined as an iCa of less than 1.15 mmol/l, which is reported to occur in 55–88.5% of critically ill patients [4,7,25–29], making it a focus of research interest.

Indeed, hypocalcaemia appears to be correlated to several well known markers of disease severity in the critically ill, including sequential organ failure assessment (SOFA) score, acute physiological and chronic health evaluation (APACHE) score, lactate and albumin levels [1,20,21,23,25,28]. A post hoc analysis of the ATN trial found that moderate hypocalcaemia (iCa <1.02 mmol/l) was independently associated with the occurrence of hypotension during CRRT [30]. The authors speculated that lower ionized calcium values may have caused a decreased vascular tone and left ventricular function but did not mention whether the hypocalcaemia was observed prior to or during CRRT. Furthermore, no adjustment was performed in the analysis for volume status and albumin, essential contributors to arterial pressure. A prospective cohort study found critical illness polyneuropathy/myopathy more frequently in patients with protracted hypocalcaemia and sepsis [31]. Several studies report hypocalcaemia to be transient and to recover spontaneously in most ICU patients during convalescence from acute illness [20,24,25,32], while failing to normalise ionized calcium levels may be seen more frequently in nonsurvivors [25,32]. Prolonged serum hypocalcaemia could be a marker of refractory illness and sustained calcium influx into the cells. However, whether, in this case, the calcium influx contributes to apoptosis or is a response to cell damage and mitochondrial dysfunction remains to be elucidated.

Numerous other, mainly retrospective studies investigating associations between the prevalence of hypocalcaemia and survival of ICU patients have been published [20,23,26,27,33–36], and their results appear somewhat inconsistent. In univariable analyses, hypocalcaemia is frequently found to be associated with increased mortality [23,24,26], while in most studies that corrected for potential covariates, ionized hypocalcaemia only remains significantly associated with mortality in severe hypocalcaemia (iCa <0.90–1.00 mmol/l) [7,23,25,26,33–35,37]. Only the most extensive cohort study of these, investigating 15 409 mixed ICU patients in China, observed mild hypocalcaemia to be slightly associated with mortality [27]. However, mild hypocalcaemia was defined as iCa 0.90–1.15 mmol/l, which has a lower limit lower than most other studies used for defining mild hypocalcaemia [25,34,36] (M. Melchers, H.P.F.X. Moonen, T.S. Breeman, *et al.*, unpublished observation).

Nevertheless, the available evidence does not support a causal relationship between lowered calcium levels and poor outcomes in the ICU patient, but it may be seen as a marker of disease severity. It seems likely that hypocalcaemia is an adaptive response to critical illness and tissue damage instead of the cause of cell damage and dysfunction, but this requires confirmation in future studies.

## MANAGEMENT OF HYPOCALCAEMIA IN THE ICU

In the case of hypocalcaemia, the Society for Endocrinology endocrine emergency guidance advises treating the underlying condition responsible for hypocalcaemia (i.e. hypomagnesemia, vitamin D deficiency, hypoparathyroidism) and supplying oral calcium in case of mild hypocalcaemia (i.e. asymptomatic and serum >1.9 mmol/l) or to administer calcium gluconate intravenously in case of severe hypocalcaemia (i.e. serum calcium <1.9 mmol/l and/or symptomatic)[38]. Others suggest giving empiric dosing of intravenous calcium in case of an iCa less than 1.12 mmol/l in hospitalised patients to avoid severe hypocalcaemia [19]. Oral calcium supplements may be considered in patients with mild hypocalcaemia that can tolerate oral medications, but this may not be feasible in critically ill patients, as these often have impaired intestinal function or absorption [19,22^▪^].

Hypocalcaemia should be corrected with calcium chloride in patients with significant bleeding to maintain coagulation according to the European Trauma Guidelines [39,40^▪▪^]. However, this recommendation is based on low-quality evidence, possibly driven by pathophysiological mechanisms at stake and multiple studies concluding that hypocalcaemia is associated with poor outcomes in critically injured patients [35,40^▪▪^,^41^]. Only one study has reported results of associations between outcome and calcium administration in major bleeding patients [40^▪▪^,42^▪^]. This observational study in 386 patients requiring massive transfusion found that administered calcium dose adjusted to the citrate load was not associated with improved in-hospital survival [42^▪^]. These findings warrant further investigation by a randomized controlled trial (RCT) to prevent unnecessary correction of calcium levels.

Calcium administration in case of hyperkalaemia-induced electrocardiogram abnormalities to stabilize the myocellular membrane is common practise, yet no RCT has shown its efficacy to date [43]. On the basis of the rationale that calcium enhances electrical defibrillation and myocardial contractility, routine calcium administration used to be recommended in case of cardiac arrest until two RCTs published in the 80 s showed no benefit of calcium in pulseless electrical activity arrests [44^▪^]. Nevertheless, a significant increase in calcium administration during adult in-hospital cardiac arrest in the United States between 2001 and 2016 was observed, possibly due to unchanged recommendations concerning the routine use of calcium [45]. Recently, results of an RCT including 397 adults in Denmark showed no beneficial effects of calcium administration on the return of spontaneous circulation in patients with out-of-hospital cardiac arrest [44^▪^,^46^^▪▪^]. The trial was early terminated because fewer patients who received calcium had a favourable neurological outcome and quality of life at 90 days [46^▪▪^]. The authors hypothesized that calcium administration may have led to cytosolic and mitochondrial calcium overload, which may have promoted oxidative stress and activation of calcium-dependent proteolytic pathways, similar to the proposed mechanisms involved in calcium-induced cell toxicity in sepsis [7,13,14,16,47]. Their results should discourage physicians from administering calcium in case of cardiac arrest routinely. Exceptions could include specific causes of cardiac arrests, such as hyperkalaemia, severe hypocalcaemia or calcium channel blocker (CCB) intoxication. However, the incidence of these conditions appears scarce, as 1% of the cardiac arrest cases were attributed to hyperkalaemia and none to CCB intoxication or hypocalcaemia in a retrospective analysis [48].

Currently, no critical care guideline advises supplementing critical illness induced hypocalcaemia in ICU patients when asymptomatic and mild. Furthermore, an evidence-based threshold is lacking [1]. The latest systematic review dating from 2008 concluded that there is no clear evidence that parenteral calcium supplementation impacts the outcome of critically ill patients [49]. One recently published study has focused on the optimal response of serum iCa to calcium supplementation in ICU patients and called for future studies to focus on optimal calcium dosing, although there is no evidence that correction improves outcomes [3]. Other studies even conclude that parenteral calcium in critically ill patients does not adequately improve ionized calcium levels [25,41,50,51]. Despite the absence of an evidence-based threshold for calcium supplementation and its inefficacy in restoring serum calcium levels, as well as the transient nature of hypocalcaemia, calcium administration occurs frequently in the ICU. In 18–79% of the ICU patients included in observational studies, calcium was administered [7,25,29,32,52,53] (M. Melchers, H.P.F.X. Moonen, T.S. Breeman, *et al.*, unpublished observation). Given the large scale of the calcium administration in these studies, it has most likely been given in most patients as an attempt to correct hypocalcaemia, which may be driven by several older studies suggesting that calcium correction should be pursued to improve the hemodynamic status of critically ill patients by enhancing ventricular contractility and vascular tone [54,55]. Still, a long-lasting effect of calcium administration on cardiac output in critically ill humans has not been described, and there is even some evidence that it may attenuate the cardiac β-adrenergic responses [55]. In addition, observational studies in the ICU have shown that calcium administration was associated with a new onset of shock [53] and a longer time to shock resolution (M. Melchers, H.P.F.X. Moonen, T.S. Breeman, *et al.*, unpublished observation), alongside higher rates of inotropic use in those that normalized calcium levels following supplementation [3]. Collage *et al*. [7] concluded that vascular permeability in septic mice increases after a single dose of calcium administration via calcium/calmodulin-dependent protein kinase signalling, which may further lead to an increased risk of organ dysfunction and death. They report the highest increase of vascular permeability in the lungs, which could explain the association between calcium administration and a reduction in ventilator-free days in their human ICU cohort [7]. Other studies examining septic animal models also concluded that parenteral calcium administration adds up to the inflammation and cell damage induced high intracellular calcium, contributing to the activation of destructive enzymes, mitochondrial dysfunction and cell death, which may worsen organ dysfunction and survival rates [13,14,16,47].

These conclusions are supported by increasing evidence about prior use of CCBs being protective in sepsis patients. A recently published meta-analysis concluded that preadmission use of CCBs was associated with a reduced 90-day mortality rate in sepsis patients and a reduced short-term mortality rate in septic shock patients [56^▪^]. There are several proposed protective mechanisms underlying this association, including the inhibition of pro-inflammatory factors, muscle proteolysis and immunosuppression by CCB, but most importantly, the inhibition of excessive calcium influx into the cell and, as a result of this reduction of cellular toxicity, attenuation of further tissue damage and organ dysfunction [56^▪^,^57^,^58^].

No RCT has assessed the risks and benefits of calcium supplementation in critically ill hypocalcaemic patients. However, several observational studies have been published (Table 1). The most extensive one by Zhang *et al.*[52] in mixed ICU patients found calcium supplementation to be independently associated with better 28-day survival. However, the proportion of sepsis patients and the method of calcium supplementation were not reported. In a subset of this cohort of sepsis patients, He *et al*. [29] found a longer ICU and hospital length of stay (LOS) after calcium supplementation was given. Furthermore, a higher 90-day mortality was found following calcium administration in patients with iCa 1.01–1.20 mmol/l, while in patients with iCa levels below 1.01 mmol/l, it might be beneficial [29]. These findings align with results from our observational study that septic patients with iCa between 1.06 and 1.14 mmol/l when calcium was administered had worse long-term survival rates (M. Melchers, H.P.F.X. Moonen, T.S. Breeman, *et al.*, unpublished observation). Most iCa levels of patients appear to be within this range during ICU admission, and most patients in the ICU suffer from inflammation to some extent, which may explain why other studies reveal that calcium administration is associated with adverse effects [7,53,59]. In some studies, this association is more pronounced with increased calcium supplementation [53,59].

**Table 1:** Summary of studies investigating the associations between calcium administration and outcome parameters in adult critically ill patients

Ref.	Country	Design	Patients	Type of patients	Hypocalcaemia prevalence	Calcium administration	Main findings
Steele *et al.*[25]	United Kingdom	Retrospective observational	1038	Mixed ICU	55%	78% received calcium gluconate parenterally.	Calcium administration was not associated with mortality or return of serum calcium to normal values.
Collage *et al.*[7]	United States	Retrospective observational	526	Sepsis	72%	18% received calcium gluconate or calcium chloride parenterally.	Calcium administration was associated with increased mortality, increased risk of renal dysfunction and reduced VFD.
Zhang *et al.*[52]	China	Retrospective observational	32 551	Mixed ICU	NR	33% received administration (type not reported). Median calcium intake was 13.9 mmol.	Calcium supplementation was associated with improved 28 and 90-day mortality.
Dotson *et al.*[53]	United States	Retrospective observational	259	Mixed ICU receiving PN	80%	79% received calcium gluconate parenterally.	Calcium administration was associated with mortality, acute respiratory failure and new onset shock. The odds of adverse outcomes increased as the calcium dose increased.
He *et al.*[29]	China	Retrospective observational	5761	Sepsis	70%	47% received administration (type not reported).	Calcium administration was associated with higher hospital mortality when iCa levels were 1.01--1.20 mmol/l. Calcium supplementation was associated with worse outcomes when the SOFA score was ≤ 4 but beneficial when the SOFA score was ≥ 8 and in case of severe hypocalcaemia (iCa <1.01 mmol/l).
M. Melchers, H.P.F.X. Moonen, T.S. Breeman, *et al.* unpublished observation)	Netherlands	Retrospective observational	1320	Mixed ICU	83%	52% received calcium gluconate parenterally with a median dosage of 2000 mg	Calcium administration was associated with a longer time to shock resolution and decreased 180-day survival in patients with sepsis and mild hypocalcaemia (iCa 1.06–1.15 mmol/l).

One study was not included in the table concerning an abstract of a retrospective observational study including 792 patients, of whom 64 received inappropriate intravenous calcium. The authors concluded that intravenous calcium did not improve iCa levels, but no association with outcome was reported [51]. iCa, ionized calcium; ICU, intensive care unit; LOS, length of stay; PN, parenteral nutrition; SOFA, sequential organ failure assessment; VFD, ventilator-free days.

Aside from direct supplementation of calcium, there has been increasing interest in the supplementation of vitamin D in patients admitted to the ICU. A recently published systematic review including 16 RCTs concluded that vitamin D administration in critically ill patients might positively affect clinical outcomes but still requires confirmation by a more extensive trial [60^▪^]. The efficacy of vitamin D supplementation appears to be more robust when parenterally administered and in patients with severe deficiency [60^▪^,^61^]. However, its effects may more likely be attributable to the immune-modulating functions of vitamin D rather than the recovery of calcium homeostasis in the critically ill. The VITdAL-ICU trial found only a difference in iCa levels at 6-month follow-up between the vitamin D supplemented group and placebo. In contrast, no differences in serum iCa on days 3, 7 or 28 were found [61].

## CONCLUSION

Serum hypocalcaemia is frequently encountered among ICU patients due to several aspects of critical illness and, to a lesser extent, iatrogenic causes. Results from observational studies are inconsistent concerning the prognostic value of hypocalcaemia, but those patients with prolonged and/or severe hypocalcaemia may be at risk of poor outcomes. Ionized hypocalcaemia may reflect a high intracellular calcium concentration, which might instead be a marker of cell damage and disease severity than the cause of clinical deterioration. Furthermore, there appears to be no clear evidence that correction of hypocalcaemia improves clinical outcomes, while some studies suggest it may provoke harm to ICU patients. Prospective studies are warranted to assess the risks and benefits of calcium administration in hypocalcaemic ICU patients and to elucidate the pathophysiological mechanisms involved. In contrast, studies assessing the optimal calcium dosing for supplementation should be discouraged until more evidence is available.

## Acknowledgements

*None.*

### Financial support and sponsorship

*None.*

### Conflicts of interest

*Prof. Dr A.R.H. van Zanten reported receiving honoraria for advisory board meetings, lectures, research and travel expenses from Abbott, AOP Pharma, Baxter, Cardinal Health, Danone-Nutricia, Dim-3, Fresenius Kabi, Mermaid, Lyric and Nestle-Novartis. The other author has nothing to declare.*

## REFERENCES AND RECOMMENDED READING

Papers of particular interest, published within the annual period of review, have been highlighted as:

▪ of special interest
▪▪ of outstanding interest
